# Trace Elements and Persistent Organic Pollutants in Unhatched Loggerhead Turtle Eggs from an Emerging Nesting Site along the Southwestern Coasts of Italy, Western Mediterranean Sea

**DOI:** 10.3390/ani13061075

**Published:** 2023-03-16

**Authors:** Mauro Esposito, Silvia Canzanella, Doriana Iaccarino, Angela Pepe, Fabio Di Nocera, Teresa Bruno, Laura Marigliano, Donato Sansone, Sandra Hochscheid, Pasquale Gallo, Fulvio Maffucci

**Affiliations:** 1 Istituto Zooprofilattico Sperimentale del Mezzogiorno, 80055 Portici, Italy; 2 Centro di Referenza Nazionale per l’Analisi e Studio di Correlazione tra Ambiente, Animale e Uomo, IZS Mezzogiorno, 80055 Portici, Italy; 3 Marine Turtle Research Group, Department of Marine Animal Conservation and Public Engagement, Stazione Zoologica Anton Dohrn, Via Nuova Macello 16, 80055 Portici, Italy

**Keywords:** *Caretta caretta*, sea turtles, POP_S_, trace elements, eggs

## Abstract

**Simple Summary:**

The western Mediterranean is an important nesting area for sea turtles, but at the same time, a hotspot for human-induced threats. Persistent organic pollutants (POPs) such as polychlorinated biphenyls (PCBs) and organochlorine pesticides (OCPs), as well as toxic and potentially toxic elements, are insidiously and ubiquitously distributed in the marine environment. These contaminants can accumulate in tissues, organs, and fluids of loggerhead turtle *Caretta caretta*, and maternal transfer of these chemicals via egg yolk during reproduction may affect the reproductive success of nesting populations. In this study, the levels of organochlorine pesticides, six indicator polychlorinated biphenyls, and trace elements were measured in unhatched eggs of *C. caretta.* With the exception of organochlorine pesticides, these contaminants were detected in all samples tested, demonstrating the transfer of chemicals from mothers to their progeny. However, their concentrations did not influence reproductive parameters. This study confirms the use of turtle eggs as a pollution monitoring tool and contributes to the scientific knowledge on the effects of environmental changes and human activities on sea turtle populations needed for the conservation of the species.

**Abstract:**

Marine pollution is one of the major threats affecting loggerhead turtles, which due to their long life span, highly migratory behavior, and carnivorous diet, may be exposed to elevated levels of toxic elements throughout their life. The transfer of chemicals from mothers to their offspring is of particular conservation concern because it may affect embryonic development and hatching success. In this study, the concentrations of 16 toxic and potentially toxic trace elements, 6 indicator polychlorinated biphenyls (PCBs), and organochlorine pesticide residues (OCPs) were determined in 138 eggs from 46 loggerhead turtle nests laid during the 2021 nesting season in Campania, Italy, western Mediterranean Sea. The possible impact of pollutant levels on hatching success and early embryonic death was also investigated. Trace element analysis was performed using an ICP-MS, except for mercury, which was determined using a Direct Mercury Analyzer® (DMA). PCBs and OCPs were analyzed with high-resolution gas chromatography coupled with high-resolution mass spectrometry (HRGC-HRMS) and gas chromatography tandem quadrupole mass spectrometry GC-MS /MS, respectively. The concentrations of essential elements in the eggs were higher than those of non-essential elements. In addition, the highly chlorinated PCBs (153, 138, and 180) contributed the most to the total PCBs, while OCPs were not detected. No correlations were found between contaminant concentrations and reproductive parameters (hatching success and no obvious embryos). The results obtained suggest that the levels of contaminants found in the eggs do not affect the reproductive success of the species in the study area.

## 1. Introduction

The loggerhead turtle (*Caretta caretta* Linnaeus, 1758) is the most common and widely distributed sea turtle species in the Mediterranean Sea [1]. Historically, nesting was confined to the warmer eastern basin, but during the last decade, the species has started to expand its nesting range into the western Mediterranean Sea, taking advantage of the increased habitat suitability caused by climate warming [2,3,4,5]. Nests are still quite scattered, but a few locations, particularly along the coasts of Campania, southwestern Italy, have now emerged as regular although minor nesting sites [3,4].

Following more than three decades of conservation efforts, mainly at major rookeries, the Mediterranean loggerhead turtle sub-population has been classified as “Least Concern” under the IUCN Red List criteria in the latest assessment [6]. However, this status is conservation-dependent due to the persistence of significant threats such as fishery bycatch, marine and terrestrial habitat degradation, climate change, and marine pollution [7]. In particular, our knowledge of chemical pollutants in the loggerhead turtle is still not adequate, and assessing the concentrations and effects of these substances has been listed among the top research priorities [7,8].

Persistent organic pollutants (POPs), such as polychlorinated biphenyls (PCBs) and organochlorine pesticides (OCPs), as well as toxic and potentially toxic trace elements, are insidious and ubiquitous chemicals distributed in the marine environment [9].

Organochlorine pesticides (OCPs), including endosulfan (α, β, and sulfate), chlordane (cis and trans) α, β, and γ (lindane)-hexachlorocyclohexane (HCH), hexachlorobenzene (HCB), dichlorodiphenyltrichloroethane (DDT) and metabolites (DDD, DDE), aldrin and heptachlor, are synthesized artificially and are used mainly against insects and other pests. These extremely toxic substances act as environmental endocrine-disrupting chemicals in humans and animals. While OCPs are largely restricted or banned in developed countries, large-scale production continues in developing countries because they are relatively cheap and easy to produce [10].

Polychlorinated biphenyls (PCBs), a synthetic group of chemicals used as plasticizers, heat-exchanging fluids, lubricants, additives in pesticides, and other industrial applications, are also of great environmental concern. Although PCBs use and production was banned in 1979, their occurrence in the environment and their bioaccumulation in the food chain are ubiquitous due to low elimination rates and high resistance to metabolic degradation [11]. PCBs are a cause of concern because chronic exposures of animals and humans to these chlorinated contaminants, even at low concentrations, alter the normal functioning of the immune, endocrine, and reproductive systems [12,13].

POPs have been detected at relatively high concentrations in several wildlife species at different levels of the food chain, and they have also been found in remote areas due to long-range atmospheric transportation [9,14,15,16].

Trace elements, i.e., metals and metalloids, occur naturally in seawater, and some of them are essential at low concentrations for animal wellbeing but become toxic at high levels. On the other hand, heavy metals such as lead, cadmium, and mercury have no known beneficial properties and are highly toxic, even at low concentrations. Trace element sources are both natural (volcanic activity, erosion of sediments) and anthropogenic (urban discharges, fertilizers or sewage sludge in agriculture, mining and smelting of sulfide ores, fuel combustion, industrial waste) and monitoring their concentrations in the biota and the environment is important to promptly identify situations where they might cause biological harm [17].

Loggerhead turtles are exposed to the risk of contamination by chemical pollutants through food, water, and sediments. The diet of these carnivorous animals varies during their lifetime. Early juveniles feed predominantly on epipelagic animals, while later juveniles and adults prefer benthic prey, although they possess high foraging flexibility and may opportunistically feed on the available food resources both in the water column and on the sea bottom [18]. Variable concentrations of polychlorobiphenyls, dioxins, organochlorinated pesticides, and toxic and potentially toxic elements have been found in tissues, organs, and fluids of this species, but their effects on health parameters are still not sufficiently understood [12,19,20,21,22,23,24,25,26]. Contaminant levels in nesting females and eggs are of particular concern because of their potential impact on embryonic development, hatching success, and early life phases [27,28,29,30,31,32,33]. Concentrations of POPs and toxic elements in sea turtle eggs are indicative of contamination on the foraging grounds of adult females [34,35,36]. Loggerhead turtles are, in fact, capital breeders that invest energy acquired prior to the nesting season in egg production [37]. Therefore, females foraging at the same site would most likely produce eggs with comparable concentrations of persistent contaminants irrespectively of their nesting site [38].

In the Mediterranean Sea, the vast majority of the studies focused specifically on the presence of persistent organic pollutants (POPs) such as PCBs, PAHs, and DDT metabolites or trace elements in tissues and organs of stranded juveniles and adults [12,19,21,39,40,41,42,43,44,45,46,47]. Very little is known about levels of contaminants in eggs, the pattern of maternal transfer of these pollutants, and their potential effect on the reproductive success of nesting populations [31,48]. Detectable concentrations of heavy metals and organochlorine contaminants were found in loggerhead turtle whole eggs (yolk and albumen) collected at nesting beaches in Cyprus, but the very small sample size (three eggs analyzed for trace elements and only one for organic pollutants) did not allow a detailed analysis [49,50]. Trace elements, namely iron, zinc, copper, cadmium, lead, and mercury, have also been analyzed in 22 samples of eggshells and remaining yolks collected at four nesting beaches in Turkey, with mercury being the only toxic element always below the detection limit [48]. More recently, the occurrence of 18 heavy metals was investigated in six eggs from three different nests laid on Linosa Island, an Italian minor nesting site located in the eastern Mediterranean, and five elements (tin, nickel, barium, antimony, lead, and chromium) were below the detection limit [51].

To the best of our knowledge, no information is available on the levels of organic and inorganic pollutants in loggerhead turtle eggs from the nests laid in the western Mediterranean Sea, where loggerhead turtles have already expanded their nesting range to the southwestern Italian coasts [3].

The aims of the present study were to (1) provide baseline concentrations for several contaminants, including toxic and potentially toxic trace elements, PCBs, and OCPs, in loggerhead turtle eggs from western Mediterranean nesting sites; (2) contribute to a better understanding of the maternal transfer of these chemicals to eggs; and (3) investigate the possible relationships between contaminant concentrations, hatching success and early embryonic death.

## 2. Materials and Methods

### 2.1. Sample Collection and Reproductive Parameters

**Figure 1:**
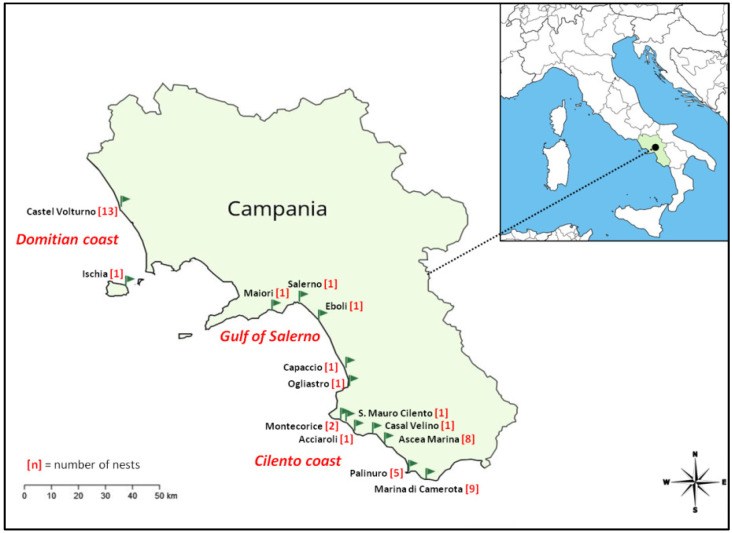
Map showing the geographical locations of sites in the Campania region, southern Italy, where nests were sampled.

Samples were collected during the 2021 nesting season from nests laid along the coasts of the Campania region, southwestern Italy (western Mediterranean Sea, Figure 1) by authorized personnel of the Marine Turtle Research Group of the Stazione Zoologica Anton Dohrn, which coordinates the regional marine turtle monitoring network (permit number PROT. No. m_amte.PNM.REGISTRO UFFICIALE.U.0000992 del 22-01-2020 issued by the Italian Ministry of Ecological Transition, MiTe). This area has been recently identified as a hotspot for loggerhead turtle nesting range expansion in the western Mediterranean and hosts more than 50% of the nests laid each year in this basin [3].

Nests were located either during the systematic beach patrols conducted on selected coast sectors or thanks to private citizens who reported the presence of females, hatchlings, or their tracks on the beach.

Nest excavations were conducted at the end of the emergence phase to determine the clutch size and hatching success (HS), which is defined as the proportion of eggs from which hatchlings emerge in the nest chamber [52].

Three to five whole unhatched eggs from each nest were collected in resealable plastic bags and stored at −20 °C for the successive analysis.

The remaining unhatched eggs were opened on-site to define the embryonic developmental stage following Miller et al. [53]. Eggs without a visible embryo are undeveloped eggs due to early embryonic mortality or infertility [53].

### 2.2. Sample Preparation

Once in the lab, eggs were slowly thawed at room temperature, then washed with distilled water to remove any sand particles and closely inspected to detect lesions on the eggshell, either due to parasites or mechanical damage, that may have caused the alterations of egg contents. Only intact eggs were used for the analysis.

The eggshell was gently opened and staged to determine the extent of embryonic development following Miller et al. [53]. For each nest, three eggs, in which embryonic development had stopped at very early stages (<stage 16), were selected and polled together for contaminant analysis; only the albumen and yolk were used for analysis.

Composite egg samples were then stored at −20 °C until processing.

### 2.3. Trace Element Analysis

#### 2.3.1. Analysis of Metals and Metalloids

For trace element analysis, 0.75 g of each composite egg sample was placed in a borosilicate glass test tube and subjected to acid mineralization with 5.0 mL of 70% nitric acid for trace element analysis, 2.5 mL of 30% hydrogen peroxide, and 2.5 mL of ultrapure water, by means of a Milestone Ultrawave Microwave digestion system (FKW, Torre Boldone, Italy). The test tubes were cooled at room temperature and the samples were quantitatively recovered in 50 mL polypropylene (PP) conical centrifuge tubes and then made up to 25 mL with ultrapure water.

Trace elements were analyzed using Inductively Coupled Plasma Mass Spectrometry (ICP-MS) mod. NexION 350X (PerkinElmer, Waltham, MA, USA). As an internal standard, rhodium at a concentration of 200 ng mL^−1^ was added to standard and sample solutions by means of online mixing.

Standard solution of arsenic (As), beryllium (Be), cadmium (Cd), chromium (Cr), cobalt (Co), Gallium (Ga), manganese (Mn), mercury (Hg), molybdenum (Mo), nickel (Ni), lead (Pb), copper (Cu), iron (Fe), rubidium (Rb), selenium (Se), strontium (Sr), thallium (Tl), uranium (U), vanadium (V), and zinc (Zn) at 1000 mg L^−1^ were obtained from Perkin Elmer (Waltham, MA, USA). Nitric acid 70% (*v*/*v*) and hydrogen peroxide 30% (*v*/*v*) were obtained from Romil Ltd (Cambridge, UK), high-purity deionized water (resistivity of ca. 18.2 MΩ cm) was produced in-house by means of an Arium® pro purification system (Sartorius, Göttingen, Germany).

#### 2.3.2. Total Mercury Analysis

Total Hg was determined in composite egg samples using combustion atomic absorption spectrometry with gold amalgamation using a Direct Mercury Analyzer® (DMA 80 evo, Milestone, Sorisole, Italy). For analysis, 0.1 g of the sample was weighed directly into nickel boats and transferred to DMA–80. Total Hg concentrations were obtained by interpolation of absorbance (λ = 253.7 nm) in external analytical curves. Calibration curves were obtained by analyzing solutions of Hg at different concentrations (0.005 mg L^−1^–5 mg L^−1^) prepared by dilution of a 1000 ± 2 mg L^−1^ standard solution of inorganic mercury (Merck KGaA, Darmstadt, Germany) using ultrapure water (resistivity < 18.2 MΩ cm).

### 2.4. PCB Analysis

For the PCB analysis, 2.0 g of composite egg sample were weighed and spiked with a standard solution containing the ^13^C labeled internal standard (IS) of six NDL-PCB congeners (IUPAC 28, 52, 101, 138, 153, and 180, Cambridge Isotope Laboratories, Massachusetts, MA, USA), for the determination of compound recoveries. The sample extraction was carried out using diethyl ether (Carlo Erba Reagents, Milan, Italy) for 24 hours; then, the extract was filtered through Whatman filter paper with anhydrous sodium sulfate, and the solvent was evaporated to dryness with the rotary evaporator. The cleanup procedure was performed by means of acid diatomaceous earth columns (Merck KGaA, Darmstadt, Germany) and Florisil cartridges SPE (Biotage, Uppsala, Sweden).

PCBs were analyzed using high-resolution gas chromatography coupled with high-resolution mass spectrometry HRGC-HRMS (DFS Magnetic Sector, Thermo Fisher Scientific, Waltham, MA, USA), and their concentrations were determined with the isotope dilution method. Samples were injected in splitless mode, and the inlet and transfer line temperatures were set at 280 °C and 290 °C, respectively. The six NDL-PCB were separated on an HT 8 (60 m × id 0.25 mm × 0.25 µm, SGE Analytical Science, Victoria, Australia) fused silica capillary column coated with 8% phenyl polycarborane siloxane, and using 99.9999% pure helium as the carrier gas at a constant flow rate of 1.0 mL min^−1^. Data acquisition was performed using multiple ion detection analysis (MID), in which two isotopic masses were monitored for each NDL-PCB congener to be measured.

Quantification of individual PCB congeners (IUPAC PCB 28, 52, 101, 138, 153, and 180) was performed using the relative response factors generated from the calibration curve of reference standards. Results were expressed as µg kg^−1^ on a wet weight (w.w.) basis and on a lipid basis (lb) and were reported in Upper Bound, whereby all values below the limit of quantification (LOQ) were set to be equal to the respective LOQ. The sum of six congeners was also calculated and expressed as ΣPCBs.

### 2.5. OCP Analysis

Residues of OCPs were determined by capillary gas chromatography tandem quadrupole mass spectrometry (GC-MS/MS) after extraction and purification of egg pool samples using the QuEChERS technique.

The extraction was performed using an extraction tube Agilent Bond Elut containing 6.0 g magnesium sulfate and 1.5 g sodium acetate. Purification of extracts was obtained using a cleanup tube Agilent Bond Elut containing 1200 mg magnesium sulfate, 400 mg PSA, and 400 mg C18.

Instrumental analysis was conducted with a GC system (mod. 7890B, Agilent Technologies, CA, USA) coupled with a Triple Quad detector (mod. 6495, Agilent, CA, USA). The GC separation was obtained using a fused silica capillary column (30 m × 250 mm × 0.25 μm) 5MS Ultra Inert Agilent and was performed with a 1 μL injection into an inlet operated in splitless mode. Data analysis was performed with Agilent MassHunter software (Santa Clara, CA, USA).

Helium was used as gas at 1.0 mL min^−1^. The GC oven initial temperature was 60 °C, held for 1 min, then ramped at 40 °C min^−1^ to 120 °C, then ramped at 5 °C min^−1^ to 310 °C, for a total run time of 50 min. The MSD transfer line temperature was 300 °C. The triple quadrupole mass detector operates in negative EI mode at 70 eV with a source temperature of 280 °C.

Organochlorine pesticide mix containing dichlorodiphenyltrichloroethane and its metabolites (DDTs), endosulfan isomers, aldrin, dieldrin, endrin, hexachlorocyclohexane isomers (α, β, and γ-HCH), heptachlor and heptachlor epoxide, chlordane (cis and trans isomers), and oxychlordane, methoxychlor, and hexachlorobenzene (HCB) at 10 mg L^−1^ in acetonitrile was purchased from LabService (Bologna, Italy). This mix solution was used to obtain matrix-matched calibration curves. These calibrations curves were obtained by spiking five concentration levels (0.0025, 0.005, 0.010, 0.025, 0.050 mg L^−1^) of the analyte with the internal standard (0.030 mg L^−1^) into sample extracts and by plotting peak areas versus analyte concentrations.

### 2.6. Quality Control and Quality Assurance

Quality assurance and quality control (QA/QC) of the analyses were assessed using control samples, including process blanks, spikes, and replicates according to the procedures and precautions implemented in order to ensure the reliability of the results in accordance with UNI CEI EN ISO/IEC 17025 (2017). In addition, QC includes participation in proficiency tests and inter-laboratory studies, achieving z-scores always within the range ±2.

Quality assurance was verified through measurement of the certified reference material (ERM®-CE278k mussel tissue) provided by the European Union Joint Research Center (JRC-IRMM) in Geel, Belgium. Moreover, blank chemical determinations were performed periodically; these were run in each batch of samples in order to check the purity of reagents and to exclude possible laboratory contamination or interference in the whole analytical procedure. 

### 2.7. Statistical Data Analysis

All data were reported as arithmetic (AM) and geometric means (GM), standard deviation (SD) (Table 1 and Table 2), median, first and third quartiles (Q1 and Q3), minimum and maximum (min and max) (Tables S1 and S2). Only the detected compounds were considered to calculate the total values for a contaminant class.

The distribution of the data was tested by means of the Shapiro–Wilk test, which revealed that most variables were not normally distributed except for clutch size and concentration of some trace elements (As, Cd, Cu, Fe, Ga, Pb, Se, Zn).

The non-parametric Spearman’s rank correlation test was used to examine the correlations between contaminant concentrations (PCBs and trace elements) and reproductive parameters (hatching success and % of unhatched eggs with no visible embryo).

For all tests, a *p*-value of ≤0.05 was used to detect significant differences. Statistical analysis was performed with IBM SPSS statistics 22 software (SPSS, Inc; Chicago, IL, USA).

## 3. Results and Discussion

### 3.1. Reproductive Parameters

During the 2021 nesting season, a total of 57 loggerhead turtle nests were detected on Campanian beaches, mainly on the Cilento and the Domitian coasts in the southern and northern sectors of the region, respectively, where new, regular, although minor nesting sites have been recently identified (Figure 1) [3]. A total of 46 of the nests were sampled for contaminant analysis, collecting a minimum of 3 unhatched eggs from each. To our knowledge, this is the largest dataset ever analyzed for metals and metalloids, polychlorobiphenyls, and organochlorine pesticide concentrations in loggerhead turtle eggs.

Details on beach location and nest data are provided in Table 1. Clutch size ranged from 43 to 127 eggs (mean ± sd = 83 ± 21 eggs). Hatching success varied from 0% to 96% (mean ± sd = 72 ± 26%). Two nests failed completely to hatch; one (N-03) was repeatedly inundated by seawater during the initial phase of embryonic development before it could be relocated, while the second (N-14) was flooded by rain during the second half of the incubation period. The proportion of unhatched eggs with no visible embryo ranged from 0% to 87% (mean ± sd = 15% ± 19). We have considered these indicative of early embryonic death, given the very high fertilization rate in the loggerhead turtle [54]. However, we cannot exclude that few of them were unfertile eggs since it is very difficult to discriminate between these two categories at the time of nest excavation, after approximately 2 months of decomposition [54]. Hatching success and the proportion of unhatched eggs with no visible embryo were strongly correlated (Spearman’s *r_s_* = −0.777, *p*-value < 0.01). A total of 24 out of the 46 analyzed nests were relocated by authorized personnel within 24 hours from the deposition because they were at high risk of flooding. This conservation practice is widely employed in the case of doomed nests, but it is not without risk because it may result in higher movement-induced mortality during the very early developmental stages [55]. No statistical difference was found in the proportion of unhatched eggs without visible embryos between natural and relocated nests (*U* = 249.5; *p* = 0.5926), although the latter had a statistically significant lower median hatching success rate (89.2% and 77.3% median value respectively, *U* = 162.5, *p* = 0.0268).

### 3.2. Inorganic Pollutants in Eggs

Trace element concentrations were above the detection limit in all samples (Table 2) and decreased in the following order: Sr > Zn > Fe > Cu > Mn > > As ≅ Se > Rb > Ga > Cr > Ni > V > Pb > Hg > Co > Cd.

Strontium exhibited the highest concentrations in loggerhead turtle composite egg samples. Only a few studies have measured Sr concentrations in sea turtle eggs [17,25,56]. Similar levels to those found here have been reported from loggerhead turtle eggs collected along the eastern coast of South Africa, Indian Ocean (median value 41 mg kg^−1^ w.w.) [17], which are significantly higher than those reported from Cape Verde (median value 3.89 mg kg^−1^ w.w.), one of the major loggerhead turtle rookeries in the Atlantic Ocean [25]. Sr appears to reach even higher concentrations in leatherback turtle eggs with values above 90 mg kg^−1^ w.w. reported from the southwestern Caribbean in the Atlantic Ocean, but the reason for this interspecific difference in Sr accumulation is still not understood [17,56].

Arsenic, another non-essential element, was found at relatively high concentrations (median value 1.1 mg kg^−1^ w.w.), comparable to those measured in loggerhead turtle eggs sampled in South Africa [17]. Arsenic is known to accumulate in loggerheads, especially in the muscle tissues [19,21,57]. Concentrations at least one order of magnitude higher than those measured here were found in the muscle of juveniles and adults stranded along the southwestern and southeastern Italian coasts [19,21]. These high accumulation levels have been related to the loggerhead turtle diet, which, at least during neritic foraging, is rich in mollusks and crustaceans that contain significant amounts of arsenic [19,21]. It has been hypothesized that the majority of the accumulated As in loggerhead turtle tissues is in its organic forms (arsenobetaine and arsenocholine), which are relatively non-toxic and physiologically inactive [19,21,24]. However, arsenic speciation in sea turtle eggs still needs to be investigated and deserves particular attention from a toxicological perspective.

Lead, mercury, and cadmium were the less abundant toxic elements in the composite egg samples (Table 2). Overall, the concentrations found here were at the lower end of the range reported in the literature for loggerhead eggs [17,48,49,58]. Low Pb accumulation has been recently reported in juvenile and adult loggerheads from the south Italian coasts, which has been linked to the significant reduction in lead pollution in the Mediterranean Sea due to the limitations of Pb additive used in gasoline enforced by the European Community since 1976 [19,21].

Mercury was also among the less abundant elements found in tissues and organs of juvenile and adult loggerheads stranded along the south Italian coasts, which most likely reflects the low contamination level in their prey. However, Hg concentrations in the composite egg samples were still at least one order of magnitude lower than those found in the kidney and liver of stranded individuals from the same area [19,21,44].

Unlike Pb and Hg, cadmium has been shown to accumulate in the renal tissue of Mediterranean loggerhead turtles, probably as a result of chronic exposure to this contaminant via food [19,21,44,51]. Despite this, Cd concentrations in the composite egg samples (median value = 0.005 mg kg^−1^ w.w.) were the lowest among the analyzed trace elements. Previous studies that have quantified cadmium in loggerhead turtle egg samples reported concentrations at least 2 × higher than those found here [17,48,49,51,59].

Overall, our results showed that in the loggerhead turtle, the maternal transfer of these toxic heavy metals during egg production is low.

Essential elements such as Se, Cu, Zn, Fe, Mn, and Ni play important physiological roles, and their presence is vital for embryonic development, which explains why they are usually found at higher concentrations than toxic ones. Females must transfer into the eggs at least the minimum amount required for biological reactions, but these elements can also have adverse health effects or even be toxic to organisms at high concentrations [60].

Zinc was the most abundant essential element in our samples (median value = 29.4 mg kg^−1^ w.w.), which is consistent with information reported in the literature and reflects its important role in yolk development [61]. Zn levels in the egg content of different sea turtle species in various studies around the world are highly variable, ranging from 10 mg kg^−1^ w.w. in flatback turtles [62] to 45 mg kg^−1^ w.w. in green turtles [63] and 48 mg kg^−1^ w.w. in loggerhead turtles [17].

Iron was the second most abundant essential element (median value = 20.1 mg kg^−1^ w.w.), twice the value found by Souza et al. [25] in loggerhead turtle eggs (10.1 mg kg^−1^ w.w.).

Copper concentration in composite egg samples (median value = 1.2 mg kg^−1^ w.w.) was similar to those found in loggerhead turtle eggs from Japan (1.1 mg kg^−1^ w.w.) [59] and from Brazil (1.48 mg kg^−1^ w.w.) [25] but much lower than the value found in South Africa (4.6 mg kg^−1^ w.w.) [17].

Similarly, manganese levels in loggerhead turtle eggs analyzed in Brazil [25] were half those found in southern Italy (0.53 vs. 1.18 mg kg^−1^ w.w.).

Selenium concentrations (median value = 1.2 mg kg^-1^ w.w.) were comparable to results obtained from the yolk of loggerhead turtle eggs from Linosa Island in the eastern Mediterranean Sea (mean value 4.61 mg kg^−1^ dry weight) [51] and in the egg content of loggerhead turtles from northwest Florida, USA (5.46 mg kg^−1^ dry weight corresponding to approximately 1.36 mg kg^−1^ w.w.) [58] but lower than levels measured in the yolk of green turtle eggs from Hong Kong (mean value 3.5 mg kg^−1^ w.w.) [63].

Nickel was detected for the first time in loggerhead turtle eggs from the Mediterranean Sea. In a previous study on egg samples from Linosa island, near the Sicily Channel, this element was always below the detection limit [51]. Ni level measured in this study (median value = 0.038 mg kg^−1^ w.w.) was lower than those found in loggerhead turtle eggs from northwest Florida, USA (0.56 mg kg^-1^ dry weight) [58] and from South Africa (1.9 mg kg^−1^ w.w.) [17].

To the best of our knowledge, this is the largest dataset on trace element concentrations in loggerhead turtle eggs and contributes to the definition of baseline levels for future comparison. Data shown here are, in general, comparable to those reported in the literature, but both inter- and intra-specific differences in trace element concentrations exist. 

This variability can be explained by many factors, including different element background concentrations, differences in life history, diet, age, and growth, as well as differences in pollution sources and uptake, retention, and excretion characteristics of the different elements in different species [17].

### 3.3. Organic Pollutants in Eggs

Organochlorine pesticides such as α-HCH, lindane, β–HCH, heptachlor, heptachlor epoxide, hexachlorobenzene, chlordane (cis and trans isomers), endosulfan (α. β and sulfate isomers), DDE, DDD, and DDT, aldrin, dieldrin, endrin, and methoxychlor, were under the limit of detection in all the composite egg samples analyzed.

Similar results were obtained in eggs of loggerhead sea turtles from northwest Florida, USA, where only one OCP (p.p′-DDD) was detected, and its presence was restricted to eggs from two nesting sites [58].

Polychlorinated biphenyl concentrations for eggs of loggerhead turtles are given in Table 3.

The sum of six PCB congeners in turtle eggs ranged from 16.9 to 133.3 ng g^−1^ lipids. The mean lipid content determined in egg samples was 8.8 ± 2.6 g%.

The six congeners of PCBs were detected and quantified in all composite egg samples, which suggests vertical transfer from the mother. In the snapping turtle, *Chelydra serpentina*, a positive correlation between PCB concentrations in maternal blood and eggs was found, which supports the hypothesis of maternal transfer [64].

Highly chlorinated PCBs (153, 138, and 180) contributed the most to the sum of PCBs. In particular, the congener 153 was the predominant congener in all samples, accounting for 49% of the mean ∑PCB concentration, similar to what was found in the study on nesting beaches along the southeast coast of the U.S. [27]. The presence of more chlorinated PCBs reflects the less soluble, less volatile, and less biodegradable properties of the congeners [65]. Moreover, -hexa and -hepta chlorinated PCBs were present in the highest concentrations in the liver of stranded individuals [13] and were also more abundant in eggs. The liver is involved as a reserve source for the synthesis of yolk precursor proteins and vitellogenins, which are transported through the blood to the ovary for deposition in the yolk [31,66]. Persistent organic pollutants derived from ingested nutrients or present in the liver at the time of vitellogenesis are then the likely source of contaminants in sea turtle eggs [31]. Since vitellogenesis mainly occurs during the foraging period of sea turtles, the POPs concentrations could be indicative of contamination of adult female feeding sites.

PCBs, like pesticides, belong to environmental pollutants of anthropogenic origin. Although the production and use of PCBs have ceased completely, their traces can still be found in electrical systems, in the environment, and in food. It is for this reason that PCBs have been detected in turtle tissues from the Mediterranean Sea many years after their ban [19,39,40].

### 3.4. Correlations with Reproductive Parameters

No correlation was found between hatching success and trace element or PCB concentrations (Table 4). Similarly, there was no correlation between the percentage of eggs without visible embryos and the concentration of trace elements or PCBs.

The absence of correlations between contaminant concentrations and reproductive parameters was also observed in other studies carried out on green turtles [36] and snapping turtles [67]. Several factors may affect embryonic development and hatching success, including sand granulometry, water potential, gas exchange, temperature, parasites, and predation [68,69,70,71].

Data presented here suggest that maternal transfer of contaminants is not a major issue and does not affect the reproductive success of the species in the study area.

## 4. Conclusions

The study allows us to know the levels of some organic and inorganic contaminants in the eggs of loggerhead turtles collected on the coasts of the Campania region in southern Italy, an emerging nesting area in the western Mediterranean Sea. 

Except for OCPs, trace elements and PCBs were detected in all samples tested, confirming the transfer of chemicals from mothers to their offspring. However, contaminant concentrations measured in this study did not affect reproductive parameters. Further studies of successive nesting seasons will provide more data and a more detailed picture of the potential impacts of these contaminants on reproductive parameters.

With an extensive dataset of contaminant concentrations in loggerhead turtle eggs, this study contributes to the knowledge of the effects of environmental change and human activities on sea turtle populations, as well as conservation and management measures for this nesting area.

## Figures and Tables

**Table 1:** Data on the loggerhead turtle nests sampled during the 2021 nesting season along the Campanian coast: Nest code; Nest coordinates latitude (Lat.) and longitude (Long.); Nest location (Beach); Clutch size (*n*); Hatching success (%); Proportion of unhatched eggs (%) with no visible embryo; Relocation (Rel).

Nest	Lat.	Long.	Nest Location (Beach)	Clutch Size	Hatching Success %	No Visible Embryo %	Rel.
N-01	40.134044	15.173244	Ascea (C)	111	57.3	34	No
N-02	40.98432	13.9671	Castelvolturno (D)	90	74.4	12	Yes
N-03	40.127743	15.178593	Ascea (C)	53	0	87	No *
N-04	40.126032	15.180173	Ascea (C)	120	94.1	2	No
N-05	40.061567	15.279357	Palinuro (C)	127	46.2	40	Yes
N-06	40.023777	15.325655	Camerota (C)	98	84.5	10	No
N-07	40.063323	15.278012	Palinuro (C)	122	60.3	12	Yes
N-08	41.014585	13.934977	Castelvolturno (D)	88	77.3	26	Yes
N-09	40.987647	13.964481	Castelvolturno (D)	117	56.4	0	Yes
N-10	40.12566	15.18051	Ascea (C)	103	89.2	6	No
N-11	40.978316	13.972573	Castelvolturno (D)	96	85.4	5	No
N-13	40.230402	14.959007	Montecorice (C)	116	77.4	16	Yes
N-14	40.193241	15.018917	S.Mauro Cilento (C)	62	0	10	Yes *
N-15	40.01558	15.333468	Camerota (C)	86	90.6	3	Yes
N-16	40.025687	15.323973	Camerota (C)	96	94.8	9	No
N-17	40.98723	13.96504	Castelvolturno (D)	59	27.1	58	Yes
N-18	40.063461	15.278016	Palinuro (C)	96	94.8	2	No
N-19	40.126477	15.179533	Ascea (C)	91	76.9	16	No
N-20	40.990556	13.961944	Castelvolturno (D)	69	95.7	6	No
N-22	40.999299	13.95383	Castelvolturno (D)	65	95.4	0	Yes
N-23	40.988285	13.964209	Castelvolturno (D)	54	77.8	13	Yes
N-24	40.005567	15.348314	Camerota (C)	70	31.4	39	Yes
N-26	40.229115	14.961578	Montecorice (C)	102	84.3	8	Yes
N-27	40.65988	14.79766	Salerno (S)	84	75	6	Yes
N-28	40.134726	15.172593	Ascea (C)	67	59.7	27	No
N-29	40.028213	15.321021	Camerota (C)	93	87.1	1	Yes
N-31	40.053617	15.282841	Palinuro (C)	107	96.3	0	No
N-32	40.028996	15.320148	Camerota (C)	77	37.7	34	No
N-33	40.138943	15.168128	Ascea (C)	81	11.1	77	Yes
N-34	40.169591	15.135516	Casalvelino (C)	79	91	5	No
N-35	40.00485	15.349346	Camerota (C)	74	83.8	0	No
N-36	40.9200191	14.0211638	Castelvolturno (D)	66	71.2	17	Yes
N-37	40.9190201	14.021988	Castelvolturno (D)	69	94.2	3	No
N-38	40.98829	13.96395	CastelVolturno (D)	61	52.5	28	No
N-39	40.427531	14.981157	Capaccio (S)	55	83.6	4	Yes
N-40	40.189125	15.021786	Acciaroli (C)	93	82.8	6	Yes
N-41	40.233266	14.952506	Ogliastro (C)	91	86.8	1	Yes
N-42	41.009253	13.941108	Castelvolturno (D)	49	69.4	10	Yes
N-43	40.016488	15.331952	Camerota (C)	83	84.3	5	Yes
N-46	40.497466	14.933575	Eboli (S)	43	78.6	12	Yes
N-48	40.069724	15.274997	Palinuro (C)	51	88.2	0	Yes
N-49	40.941694	14.0074959	Castelvolturno (D)	78	74.4	3	Yes
N-51	40.741606	13.864245	Ischia (I)	83	39.8	35	No
N-52	40.648808	13.63596	Maiori (S)	97	92.8	1	No
N-54	40.000393	15.367456	Camerota (C)	68	95.6	0	No
N-55	40.136656	15.170769	Ascea (C)	82	96.3	0	No

C: Cilento coast; D: Domitian coast; I: Island of Ischia; S: Gulf of Salerno. * Inundated nests.

**Table 2:** Statistical values (GM, AM, SD) of trace element concentrations found in the egg samples of loggerhead sea turtles expressed in mg kg^−1^ w.w.

Element	GM	AM	SD
As	1.07	1.13	0.35
Cd	0.005	0.006	0.003
Co	0.010	0.012	0.007
Cr	0.073	0.096	0.090
Cu	1.12	1.18	0.384
Fe	18.5	19.1	4.64
Ga	0.204	0.217	0.065
Hg	0.011	0.013	0.006
Mn	1.23	1.54	1.06
Ni	0.041	0.055	0.054
Pb	0.029	0.032	0.014
Rb	0.403	0.425	0.121
Se	1.07	1.12	0.33
Sr	36.6	38.7	11.4
V	0.035	0.038	0.015
Zn	28.3	30.0	9.58

GM: geometric mean; AM: arithmetic mean; SD: standard deviation.

**Table 3:** Statistical values (GM, AM, SD) of six PCB congeners and their sum in egg content of loggerhead sea turtles expressed as ng g^−1^ of lipid.

PCB	GM	AM	SD
28	0.24	0.26	0.089
52	0.21	0.23	0.13
101	0.41	0.45	0.21
138	10.2	11.5	6.2
153	19.9	22.7	13.5
180	9.8	11.3	7.2
Σ PCBs	40.9	46.4	26.9

GM: geometric mean; AM: arithmetic mean; SD: standard deviation.

**Table 4:** Spearman’s correlation coefficients (r_s_, *p*) measuring the strength of the association between trace element or PCB concentrations and hatching success or unhatched eggs with no visible embryos.

	Hatching Success		Unhatched Eggs	
	r_s_	*p*	r_s_	*p*
**Trace element**				
As	−0.035	0.82	0.15	0.33
Cd	−0.0003	1.0	0.018	0.91
Co	0.27	0.082	−0.15	0.34
Cr	0.25	0.097	−0.15	0.32
Cu	−0.29	0.054	−0.16	0.30
Fe	0.25	0.092	−0.13	0.38
Ga	0.12	0.42	−0.067	0.66
Hg	0.19	0.20	−0.21	0.16
Mn	−0.037	0.81	0.079	0.61
Ni	0.26	0.083	−0.20	0.20
Pb	0.023	0.88	0.011	0.94
Rb	−0.083	0.59	0.12	0.43
Se	0.25	0.10	−0.13	0.40
Sr	0.16	0.28	−0.096	0.53
V	−0.0012	0.99	0.023	0.89
Zn	0.27	0.069	−0.15	0.32
**PCB**				
PCB-28	0.15	0.33	−0.16	0.29
PCB-52	−0.017	0.91	−0.025	0.87
PCB-101	0.16	0.30	−0.094	0.53
PCB-138	0.19	0.20	−0.18	0.25
PCB-153	0.21	0.17	−0.20	0.18
PCB-180	0.12	0.44	−0.11	0.47

## Data Availability

Not applicable.

## Supplementary Materials

The following supporting information can be downloaded at: https://www.mdpi.com/article/10.3390/ani13061075/s1; Table S1: Statistical values (Min, Q1, median, Q3, Max) of trace element concentrations found in the egg samples of loggerhead sea turtle expressed in mg kg^−1^ w.w. Table S2: Statistical values (Min, Q1, median, Q3, Max) of six PCB congeners and their sum in egg content of loggerhead sea turtles expressed as ng g^−1^ of lipid.
