# Supplementary material for: Trace Elements and Persistent Organic Pollutants in Unhatched Loggerhead Turtle Eggs from an Emerging Nesting Site along the Southwestern Coasts of Italy, Western Mediterranean Sea
Source: Animals (Basel). 2023 Mar 16;13(6):1075. doi: 10.3390/ani13061075 (PMC10044507; doi:10.3390/ani13061075)
Supplement: Supplementary file 1 [file animals-13-01075-s001.zip › animals-2256710-supplementary.pdf]

Table S1: Statistical values (Min, Q1, median, Q3, Max) of trace element concentrations found in the egg samples of loggerhead sea turtle expressed in mg kg^{-1} w.w.

Element	Min	Q1	median	Q3	Max
Hg	0.0020	0.0078	0.011	0.017	0.027
As	0.39	0.86	1.11	1.31	2.01
Cd	0.001	0.003	0.005	0.008	0.015
Pb	0.009	0.022	0.030	0.040	0.071
Co	0.002	0.007	0.011	0.016	0.029
Cr	0.022	0.048	0.065	0.10	0.43
Cu	0.460	0.884	1.20	1.44	2.06
Fe	9.5	15.1	20.1	22.1	28.3
Ga	0.050	0.174	0.227	0.263	0.326
Mn	0.254	0.722	1.18	2.01	4.78
Ni	0.011	0.023	0.038	0.061	0.25
Rb	0.160	0.374	0.458	0.506	0.660
Se	0.470	0.857	1.12	1.34	1.95
Sr	14.0	32.4	40.6	46.4	54.2
V	0.012	0.028	0.034	0.047	0.081
Zn	10.4	23.4	29.4	36.8	52.4

Min: minimum value; Q_1: first quartile (25% percentile); Q_3: third quartile (75% percentile); Max: maximum value.

Table S2: Statistical values (Min, Q1, median, Q3, Max) of six PCB congeners and their sum in egg content of loggerhead sea turtles expressed as ng g^{-1} of lipid

PCB	Min	Q1	Median	Q3	Max
28	0.10	0.19	0.24	0.30	0.63
52	0.099	0.14	0.19	0.28	0.69
101	0.17	0.34	0.42	0.50	1.3
138	4.2	7.4	9.68	13.7	31.8
153	8.1	14.9	19.2	26.8	69.7
180	3.5	7.1	9.3	13.1	35.6
Σ PCBs	16.9	30.5	38.9	53.7	133.3

Min: minimum value; Q1: first quartile (25% percentile); Q3: third quartile (75% percentile); Max: maximum value
